# Mortality in HIV-Infected Alcohol and Drug Users in St. Petersburg, Russia

**DOI:** 10.1371/journal.pone.0166539

**Published:** 2016-11-29

**Authors:** Nadia S. Fairbairn, Alexander Y. Walley, Debbie M. Cheng, Emily Quinn, Carly Bridden, Christine Chaisson, Elena Blokhina, Dmitry Lioznov, Evgeny Krupitsky, Anita Raj, Jeffrey H. Samet

**Affiliations:** 1 Department of Medicine, University of British Columbia, Vancouver, British Columbia, Canada; 2 Department of Medicine, Section of General Internal Medicine, Clinical Addiction Research and Education Unit, Boston University School of Medicine/Boston Medical Center, Boston, Massachusetts, United States of America; 3 Department of Biostatistics, Boston University School of Public Health, Boston, Massachusetts, United States of America; 4 Data Coordinating Center, Boston University School of Public Health, Boston, Massachusetts, United States of America; 5 Department of Medicine, Section of General Internal Medicine, Clinical Addiction Research and Education Unit, Boston Medical Center, Boston, Massachusetts, United States of America; 6 First Pavlov State Medical University, St. Petersburg, Russian Federation; 7 North-West Regional AIDS Center, St. Petersburg, Russian Federation; 8 Bekhterev Research Psychoneurological Institute, St. Petersburg, Russian Federation; 9 Division of Global Public Health, University of California San Diego School of Medicine, La Jolla, California, United States of America; University of Florida, UNITED STATES

## Abstract

In Russia, up to half of premature deaths are attributed to hazardous drinking. The respective roles of alcohol and drug use in premature death among people with HIV in Russia have not been described. Criminalization and stigmatization of substance use in Russia may also contribute to mortality. We explored whether alcohol, drug use and risk environment factors are associated with short-term mortality in HIV-infected Russians who use substances. Secondary analyses were conducted using prospective data collected at baseline, 6 and 12-months from HIV-infected people who use substances recruited between 2007–2010 from addiction and HIV care settings in a single urban setting of St. Petersburg, Russia. We used Cox proportional hazards models to explore associations between 30-day alcohol hazardous drinking, injection drug use, polysubstance use and environmental risk exposures (i.e. past incarceration, police involvement, selling sex, and HIV stigma) with mortality. Among 700 participants, 59% were male and the mean age was 30 years. There were 40 deaths after a median follow-up of 12 months (crude mortality rate 5.9 per 100 person-years). In adjusted analyses, 30-day NIAAA hazardous drinking was significantly associated with mortality compared to no drinking [adjusted Hazard Ratio (aHR) 2.60, 95% Confidence Interval (CI): 1.24–5.44] but moderate drinking was not (aHR 0.95, 95% CI: 0.35–2.59). No other factors were significantly associated with mortality. The high rates of short-term mortality and the strong association with hazardous drinking suggest a need to integrate evidence-based alcohol interventions into treatment strategies for HIV-infected Russians.

## Introduction

Russia has one of the largest HIV epidemics in the world, with almost 1 million people infected among a population of 144 million [1]. Also one of the fastest growing HIV epidemics, the number of new cases rose from 40,000 in 2006 to over 75,000 in 2012 [2]. The epidemic is driven primarily by injection drug use, with HIV prevalence rates among people who inject drugs estimated to be as high as 37% [3]. Though significant mortality improvements have been observed globally since the advent of antiretroviral therapy in 1996, access to antiretroviral therapy among people who inject drugs remains low in many settings [4]. Reduced access to antiretroviral therapy and evidence-based interventions for people who inject drugs, specifically needle syringe access programs and opioid agonist therapies with methadone and buprenorphine, perpetuate the transmission and ongoing high mortality in HIV-infected people in Russia [4–6].

In the general Russian population, alcohol consumption is among the highest in the world [7, 8]. In Eastern Europe, almost one quarter of total disease burden is attributable to alcohol, a substantial increase since 1990 [7]. Up to half of premature deaths in Russia are attributed to hazardous drinking [9]. In other settings, including North America, heavy drug use and homelessness, but not alcohol use, have been shown to be major predictors of all-cause mortality in HIV-infected people who use substances [10–13]. Concurrent use of alcohol and opioids has been associated with mortality due to overdose among substance users in settings outside Russia [14–17]. The respective roles of alcohol and drug use in premature death among people with HIV in Russia have not been described.

In addition, social and policy factors outside of individual characteristics, such as criminalization and stigmatization, that make up the ‘risk environment’ for people who use substances in Russia shape individual health-related harms and inequalities [4, 18, 19]. Some risk environment factors (i.e. past incarceration, police involvement, selling sex, and HIV stigma) have been identified as important barriers to treatment and prevention of HIV infection among substance users in Russia [20, 21]. The associations of these individual environmental risk exposures with mortality have yet to be analyzed in Russia. Because substance use and the ‘risk environment’ are dynamic conditions that change over time, focusing on deaths occurring within a shorter period of time, specifically within 12 months of the last study assessment, increases the likelihood of measuring a mortality impact from these potentially modifiable conditions when they are active.

Thus the purpose of this study was to examine factors associated with short-term mortality in a group of HIV-infected Russians who use substances. We focused on drug and alcohol use behaviors that are potentially modifiable through treatment and prevention, as well as factors that shape the risk environment, specifically interactions with police and experiences of incarceration, stigma, and sex trade involvement.

## Materials and Methods

### Study design and study population

This was a prospective, longitudinal study using data from the HERMITAGE (HIV Evolution in Russia- Mitigating Infection Transmission and Alcoholism in a Growing Epidemic) study. HERMITAGE was a single-blinded randomized controlled trial conducted to determine whether a secondary HIV prevention intervention, Healthy Relationships Intervention [22], adapted for use in Russia, decreased unsafe sex, alcohol use, and sexually transmitted infection (STI) among HIV-infected hazardous drinkers in St. Petersburg, Russia. The study has previously been described in detail [23]. From 2007–2010, participants were recruited from HIV and substance use patient care sites. Participants completed a questionnaire at baseline, and at 6 and 12-month follow-up.

In total, 921 individuals were screened and 700 participants were enrolled. Eligibility criteria for enrollment included: 18 years of age or older, HIV infected, any anal or vaginal sex without a condom in the past 6 months, and any reported past 6 months “hazardous alcohol use” defined as National Institute on Alcohol Abuse and Alcoholism (NIAAA) hazardous alcohol use (i.e. >14 drinks/week or >4 drinks on one occasion for men <65 years of age, and >7 drinks/week or >3 drinks on one occasion for all women and men ≥ 65 years of age) [24].

### Recruitment and eligibility

Participants were recruited from inpatient and outpatient HIV and substance use treatment sites, a needle syringe access program, and via snowball recruitment in St. Petersburg, Russia. At the clinical sites, research associates approached patients, assessed eligibility, offered participation, obtained written informed consent, and conducted assessments in a private room. Participants recruited from the needle syringe access program and through “snowball recruitment” were given information on the study and referred to one of the clinical sites for eligibility assessment. Of 921 people screened, 221 were excluded: 189 were ineligible, 31 declined and one was too ill to participate. Among the 189 who were not eligible, 110 did not meet the alcohol criteria and 134 did not meet the sex risk criteria. Less common reasons for ineligibility were inability to provide names of contacts for tracking information (n = 30) or had exclusion criteria including pending legal issues (n = 17), ongoing efforts to conceive (n = 4), or inability of the research team to confirm the individual’s HIV infection (n = 2). Most participants (n = 569, 81%) had at least one follow-up visit.

After recruitment and eligibility assessment, all participants provided written informed consent. Data were collected in Russian via: (a) a face-to-face interview with a research associate (S1 and S2 Appendices) and (b) a self-administered questionnaire for depressive symptoms via Beck Depression Inventory II–Russian version [25]. Participants were compensated the equivalent of US $7 for the baseline assessment, done typically while hospitalized, and $28 for 6-month and $35 for 12-month follow-up.

### Ethics statement

Institutional Review Boards of Boston University Medical Campus in Boston, USA, and First Pavlov State Medical University of St. Petersburg, Russian Federation, approved the study.

### HERMITAGE randomized controlled trial intervention

The primary outcome of the HERMITAGE (HIV's Evolution in Russia-Mitigating Infection Transmission and Alcoholism in a Growing Epidemic) randomized controlled trial was any incident STI by laboratory test at 12-month follow-up. The HERMITAGE adapted Healthy Relationships Intervention [22] consisted of two individual sessions and three small group sessions facilitated by a team of American and Russian psychologists and physicians with addiction and HIV expertise. The focus of the sessions was on disclosure of HIV serostatus as a means to facilitate effective communication about the need for condom use with vaginal or anal sex. To correspond with the structure of the HERMITAGE Intervention a five session control program was implemented that focused on stress reduction, social support and good nutrition. Participants were assigned to either the intervention or control group using stratified-blocked randomization. The randomization procedure was stratified by gender, injection drug use, and recruitment site.

### Explanatory variables

Several potentially modifiable drug and alcohol use risk behaviors were explored as potential predictors of short-term mortality. We derived two different explanatory variables to measure alcohol consumption using daily drinking information from the 30-day timeline followback questionnaire, which was administered at each interview [26, 27]. The primary measure was hazardous alcohol use defined according to the NIAAA guidelines [24]. The secondary measure of hazardous alcohol was used previously to categorize drinking levels in Russia, defined as number of bottles of vodka equivalents per week [9, 28]. Total weekly alcohol consumption was expressed in 500 mL bottles of vodka (or alcoholic equivalent). One standard beer was taken as 0.125 and wine as 0.25 times vodka strength. This measure was recently used in a large prospective cohort study examining premature death in Russia [9]. Other explanatory variables were any injection drug use in the previous 30 days, polysubstance use in the previous 30 days, incarceration ever in the past, police involvement ever in the past, selling sex in the past 3 months, and experience of HIV-related stigma. Polysubstance use was defined as two or more substances including alcohol, heroin or other opioids, ephedrine, amphetamine or methamphetamine, cocaine or crack, cannabis and club drugs, including ecstasy and ketamine. Police involvement was a dichotomized variable that included any of the following: arrest for possession of drugs or injection equipment, having drugs planted on their person by police, or being forced to give money or exchange sex with a police officer. Because any previous interactions with the criminal justice system may represent traumatic experiences that lead to persistent psychological harm [29, 30], both incarceration and police involvement were treated as time-dependent variables representing any previous history of incarceration/police involvement where we asked about exposure “ever in the past” at baseline and updated to positive exposure category at each follow-up assessment. High stigma was defined as a score greater than 2.5 on the People Living with HIV Stigma Index, Russian edition [31].

### Covariates

Potential confounders defined at baseline included age, gender, relationship status defined as married or living with a partner, HERMITAGE study randomization group, antiretroviral therapy use, years since HIV diagnosis (dichotomized at the median), overdose history, physical component score [32], moderate-to-severe depressive symptoms by the Beck Depression Inventory-II (score > 13), and alcohol or drug treatment exposure. Physical component scores range between 0 and 100, with a higher score indicating better health. All variables were based on self-report.

The primary endpoint for this analysis was time from study enrollment to death. In this study we focus on short-term mortality, defined as those occurring within 12 months of enrollment.

Causes of death were obtained from friends or family report. The study physician investigators (NF, AW, JS) used this cause of death information to allocate each death into the following categories: liver and other gastrointestinal-related diseases, trauma/suicide (physical trauma from violence or motor vehicle crash or suicide), HIV-related conditions, drug overdose, and unknown (deaths for which there was not enough information to attribute cause or death that did not fit into one of the categories above).

### Statistical Analyses

We used descriptive statistics to characterize the study sample. Separate Cox proportional hazards models were then used to examine the relationship between each of the main explanatory variables of interest and mortality. The exact method was used to handle tied event times [33]. Preliminary unadjusted models were fit for each explanatory variable. We then fit a series of regression models in order to control for potential confounding factors. Potential confounders were selected based on the literature and clinical experience. The first adjusted model included demographic characteristics that were expected to be the strongest confounders: age, gender, and marital status. We then fit a second adjusted model that additionally controlled for antiretroviral therapy use, years since HIV diagnosis, overdose history, physical component score, moderate-to-severe depressive symptoms by the Beck Depression Inventory-II (score > 13), and alcohol or drug treatment exposure. Lastly, we fit a final fully adjusted model that additionally controlled for randomization group. The findings for the main explanatory variables were similar across the series of adjusted models, therefore the results presented focus on the final fully adjusted models for each predictor of interest. Due to the limited number of events, a single model including all explanatory variables and covariates was not fit. All of the main explanatory variables were modeled as time-dependent variables as well as the following covariates: antiretroviral therapy use, overdose history, physical component score, depressive symptoms, and alcohol or drug treatment exposure. We assessed the correlation between all pairs of explanatory variables and covariates and verified that no pair of variables included in the models was highly correlated (i.e., r>0.40). Due to the exploratory nature of the analyses, no adjustment was made for multiple comparisons. An alpha = 0.05 was considered statistically significant. AnaIyses were performed using SAS software (version 9.3; SAS Institute, Cary, NC).

## Results

### Characteristics of study population

Among 700 participants, 40 (5.7%) died. The crude mortality rate was 5.9 per 100 person years. HIV-related conditions, liver-related diseases, and overdose were the three most common causes of the 40 deaths (Table 1). The median follow-up time for all participants was 12 months (interquartile range [IQR] 12–12 months), with the longest follow-up 18 months post-enrollment. At study entry, the mean age was 30 years, 59% were male, 15% were on antiretroviral therapy, the median time since HIV diagnosis was 4 years (IQR = 1–8 years), 42% reported recent use of injection drugs, and 60% fulfilled NIAAA criteria for hazardous drinking in the prior 30 days (Table 2).

**Table 1:** Causes of death among HIV-infected Russian HERMITAGE participants over 12-months.

Cause of death category	Mortality, % (No.) (*n* = 40)^‡^
HIV-related diseases	32% (13)
Liver and other gastrointestinal-related diseases	27% (11)
Drug overdose	15% (6)
Physical trauma* or suicide	10% (4)
Other	15% (6)

*From violence or motor vehicle crash.

‡Deaths occurring within 12 months of participants’ last study interview.

**Table 2:** Baseline characteristics of HIV-infected Russian HERMITAGE participants stratified by short-term mortality (n = 700).

Characteristic	Mortality ^‡^				*p*-value^ρ^
	No*		Yes		
	94.3%	(*n* = 660)	5.7%	(*n* = 40)	
**Age, mean (SD)**	30	(5.2)	31	(5.0)	0.26
**Gender, % (n)**					
Female	41%	(268)	42%	(17)	0.81
Male	59%	(392)	57%	(23)	
**Married or living with partner**					
No	64%	(425)	57%	(23)	0.37
Yes	35%	(234)	42%	(17)	
**Current ART use**^**ρ**^					
No	84%	(555)	92%	(37)	0.15
Yes	16%	(105)	7%	(3)	
**Years since HIV diagnosis, mean (n) SD**	3.6	(4)	3.6	(5)	0.68
**3-month overdose**					
No	86%	(566)	82%	(33)	0.57
Yes	14%	(94)	17%	(7)	
**Beck Depression Inventory-II, Score > 13**^**ρ**^					
No	36%	(241)	20%	(8)	0.03
Yes	63%	(419)	80%	(32)	
**Lifetime suicidality**^**ρ**^					
No	42%	(275)	47%	(19)	0.47
Yes	58%	(385)	52%	(21)	
**Physical Component Score, mean (SD)** ^**ρ**^	9%	(45)	11%	(37)	<0.05
**Alcohol or drug treatment**^**ρ**^					
No	93%	(612)	95%	(38)	0.59
Yes	7%	(48)	5%	(2)	
**Randomization group**					
Control	49%	(327)	57%	(23)	0.33
Treatment	50%	(333)	42%	(17)	
**Hazardous alcohol use (NIAAA)** ^**ρ**^					
No drinking	19%	(126)	10%	(4)	0.09
Moderate	31%	(205)	22%	(9)	
Hazardous	50%	(329)	67%	(27)	
**Hazardous alcohol use (vodka)**^‡^					
No drinking	15%	(101)	12%	(5)	
<1 500mL bottle/week	33%	(215)	25%	(10)	0.04
1–3 500mL bottle/week	35%	(232)	27%	(11)	
3+ 500mL bottle/week	17%	(112)	35%	(14)	
**30-day injection drug use**^‡^					
No	58%	(385)	52%	21)	0.47
Yes	42%	(275)	47%	(19)	
**30-day polysubstance use**^‡^					
No	53%	(351)	52%	(21)	0.93
Yes	47%	(309)	47%	(19)	
**Past incarceration**					
No	61%	(405)	55%	(22)	0.42
Yes	39%	(255)	45%	(18)	
**Past police involvement**					
No	38%	(249)	35	(14)	0.73
Yes	62%	(411)	65%	(26)	
**3-month selling sex**^‡^					
No	86%	(568)	97%	(39)	0.04
Yes	14%	(92)	2%	(1)	
**High stigma**^‡^					
No	52%	(346)	62%	(25)	0.21
Yes	48%	(314)	37%	(15)	

‡Deaths occurring within 12 months of participants’ last study interview.

*Censored, i.e. death was not observed before loss to follow-up or end of study.

^ρ^Proportions compared using chi-square/Fisher’s exact test, means compared using t-test.

### Cox proportional hazard analyses

Unadjusted and adjusted Cox proportional hazard ratios for each of the main explanatory variables are presented in Table 3. In analyses adjusted for potential confounders, the hazardous drinking measure of > 1500 mL of vodka equivalent per week, vs. < 500 mL, was significantly associated with mortality [adjusted hazard ratio (aHR) 3.51; 95% Confidence Interval (CI): 1.48–8.29] but drinking of 500–1500 mL was not (aHR 1.35, 95% CI: 0.54–3.37). The alternate hazardous drinking measure of 30-day NIAAA hazardous drinking was also significantly associated with a higher risk of mortality (aHR 2.60; 95% CI: 1.24–5.44) but moderate drinking was not (aHR 0.90, 95% CI: 0.35–2.60). High HIV stigma appeared to be associated with lower mortality (aHR 0.52; 95% CI: 0.27–1.02), although the results were not statistically significant. We observed no significant associations between injection drug use, polysubstance use, incarceration, police involvement, or selling sex with mortality.

**Table 3:** Unadjusted and adjusted hazard ratios for mortality among HIV-infected hazardous drinkers in Russia (n = 700).

Explanatory Variable	Unadjusted Hazard Ratio (95% CI)	Adjusted Hazard Ratio* (95% CI)
**Hazardous alcohol use (NIAAA)**^‡^		
No drinking	Ref	Ref
Moderate	1.04 (0.38–2.80)	0.95 (0.35–2.59)
Hazardous	3.68 (1.80–7.55)	2.60 (1.24–5.44)
**Hazardous alcohol use (vodka)**^‡^		
No drinking	Ref	Ref
<1 500mL bottle/week	0.62 (0.23–1.70)	0.57 (0.21–1.60)
1–3 500mL bottle/week	1.74 (0.72–4.18)	1.36 (0.54–3.38)
3+ 500mL bottle/week	4.77 (2.12–10.75)	3.51 (1.48–8.31)
**30-day injection drug use**^‡^		
Yes vs. no	1.63 (0.88–3.05)	1.42 (0.72–2.77)
**30-day polysubstance use**^‡^		
Yes vs. no	1.26 (0.67–2.36)	1.18 (0.61–2.30)
**Past incarceration**		
Yes vs. no	1.28 (0.69–2.39)	1.12 (0.57–2.20)
**Past police involvement**		
Yes vs. no	1.11 (0.58–2.13)	1.28 (0.64–2.54)
**3-month selling sex**^‡^		
Yes vs. no	0.16 (0.02–1.19)	0.15 (0.02–1.13)
**High stigma**^‡^		
Yes vs. no	0.67 (0.35–1.26)	0.52 (0.27–1.01)

Ref = Reference category; CI = Confidence Interval

*Separate multivariable models for each explanatory variable adjusted for potential confounders age, gender, marital status, ART use, years since HIV diagnosis, 3-month overdose, PCS score, moderate-to-severe depressive symptoms, alcohol/drug treatment, and intervention group.

‡Time-dependent variable.

Multivariable Cox models including all the explanatory variables of interest were fit in post-hoc analyses—one model using the primary NIAAA definition and one model using the secondary variable based on the vodka equivalent definition for hazardous alcohol use. Explanatory variables of interest were included in these two models but no covariates were included due to the limited number of outcomes being examined. The conclusions for most explanatory variables remained consistent in these multivariable models except injection drug use (aHR 4.11, 95% CI: 1.12–15.07) and selling sex (aHR 0.12, 95% CI: 0.02–0.89), which achieved statistical significance in these models.

Among the covariates, physical component score (aHR 0.94 per 1 point increase in score; 95% CI: 0.91–0.97) and moderate to severe depressive symptoms (aHR 2.50; 95% CI: 1.11–5.60) were also significantly associated with mortality in the Cox proportional hazards model for hazardous drinking defined by the NIAAA (Table 4). The results for physical component score and depressive symptoms were similar across adjusted models for the other main explanatory variables of interest. No other covariates, including age, gender, marital status, history of alcohol or drug treatment, intervention group, antiretroviral therapy use, or years since HIV diagnosis were associated with mortality for the adjusted models of the other main explanatory variables of interest.

**Table 4:** Multivariable Cox proportional hazards model for hazardous alcohol use among HIV-infected Russians (n = 700).

Characteristic	Hazard Ratio* (95% CI)	*p-value*
**Hazardous alcohol use (vodka)^‡^**		
**No drinking**	**Ref**	**Ref**
**Moderate**	0.95 (0.35–2.59)	0.91
**Hazardous**	2.60 (1.24–5.44)	0.01
**Age, per year**	1.02 (0.96–1.08)	0.59
**Gender**		
**Female vs. male**	0.77 (0.39–1.53)	0.46
**Married or living with Partner^‡^**		
**Yes vs. no**	1.35 (0.70–2.63)	0.37
**3-month overdose^‡^**		
Yes vs. no	1.22 (0.53–2.82)	0.63
**Physical Component Score, per point increase**	0.94 (0.91–0.97)	<0.001
**Alcohol or drug treatment^‡^**		
Yes vs. no	0.35 (0.10–1.15)	0.08
**Randomization Group^‡^**		
Intervention vs. Control	0.69 (0.35–1.34)	0.27
**Current ART use^‡^**		
Yes vs. no	1.18 (0.27–5.13)	0.83
**Years since HIV diagnosis (>3.8 years)**		
Yes vs. no	1.06 (0.56–2.02)	0.86
**Beck Depression Inventory-II Score >13^‡^**		
Yes vs. no	**2.50 (1.11–5.60)**	0.03

Ref = Reference category; CI = Confidence Interval; ART = antiretroviral therapy

*Multivariable model adjusted for age, gender, marital status, ART use, years since HIV diagnosis, 3-month overdose, PCS score, moderate-to-severe depressive symptoms, alcohol/drug treatment, and intervention group.

‡Time-dependent variable

## Discussion

This cohort of Russian HIV-infected people who use substances had strikingly high rates of short-term mortality. The crude mortality rate of 5.9 per 100 person years was much greater than the general Russian population of comparable age where the mortality rate is 1.5 per 100 person years, with a standardized mortality ratio of 3.9 [34]. A meta-analysis investigating mortality among HIV-infected opioid users that included studies from Europe and the US also documented a lower pooled estimate mortality rate of 2.9 per 100 person years [35].

The mortality rate was also greater than in other HIV-infected cohorts in the post-highly active antiretroviral era [36]; the overall mortality rate was 1.3 per 100 person years in the Swiss HIV cohort [36] and 2.4 per 100 person years in the Danish HIV cohort study [37]. Among persons with homosexual or heterosexual HIV transmission in the Swiss HIV cohort, drug use was associated with higher all-cause mortality (subhazard rate = 1.7), compared with no drug use, but hazardous alcohol use was not associated with risk of death [36]. In the Danish HIV cohort study, the mortality rate in HIV-infected patients more than tripled when the primary route of infection was injection drug use compared with other routes of transmission (mortality rate ratio = 3.2) [37]. Compared to our study population where death from HIV-related diseases was the most common cause of death and antiretroviral uptake was low, the most frequent causes of death among the non-substance users in the Swiss HIV cohort were non-AIDS-related malignancies, cardiovascular disease, and infections (44%), followed by liver and other gastrointestinal-related diseases (9%) HIV-related diseases (8%), and physical trauma or suicide (7%) [38]. Non-HIV-related conditions were also the most common causes of death in the Danish HIV cohort [37]. Our study underscores the high mortality rate identified in our study population of people who use substances compared with other HIV-infected populations (5.9 per hundred person years in Russia vs. 1.3 and 2.4 per hundred person years in the Swiss and Danish cohorts respectively). Though increased mortality has been associated with drug use among HIV-infected populations, we identified a strong association with hazardous alcohol use. The low uptake of antiretroviral therapy among study participants likely accounts for HIV-related conditions being the most common cause of death and contrasts with other settings with higher antiretroviral uptake.

Hazardous alcohol use was independently associated with short-term mortality in this setting. This association was the dominant one and persisted, even after controlling for known important covariates, such as antiretroviral therapy use and recent injection drug use [35, 39]. Hazardous alcohol may lead to premature mortality by several mechanisms [40]. Liver disease may be accelerated, particularly in the setting of hepatitis B and/or C co-infection, a common comorbidity in people who inject drugs [41]. Trauma and suicide often occur in the setting of alcohol use. Mortality from tuberculosis, a major opportunistic infection leading to HIV-related deaths in Russia which was not measured in our study, is common among HIV-infected individuals who drink alcohol in Eastern Europe and the risk of active tuberculosis has been found to be substantially elevated in people with hazardous alcohol use [42, 43]. Hazardous drinkers have poorer HIV outcomes related to effective use of antiretroviral therapy and viral load suppression [44, 45]. Further study is needed to better understand the observed association with hazardous alcohol use among HIV-infected people in the Russian context to help understand how to address alcohol use in a way that will improve mortality.

These findings are notable because alcohol use has not consistently been shown to be associated with mortality in HIV-infected substance users in other settings. Features of alcohol use in Russia that may convey particularly high risk include predominance of spirits (i.e. vodka), high quantities of alcohol consumed, and pattern of binge drinking [46, 47]. Heterogeneity of findings across studies may also be attributed to varied methodology and measurement [45]. The vodka measure for hazardous alcohol use gives hints that there may be a threshold effect at 1500mL of vodka. This is equivalent to about 51 ounces of vodka or equivalent per week, as compared to the NIAAA maximum threshold of 21 ounces of vodka or equivalent per week [24]. Another possible explanation for inconsistent findings is that the association between alcohol use and mortality is masked in other settings by the “sick quitter” phenomenon where sicker persons are less likely to be drinkers [10, 48].

Russia has had wide fluctuations in mortality rates attributed in part to changing alcohol policies over time [47]. Temporary reductions in alcohol-related mortality were documented in 1986 with the anti-alcohol campaign led by Mikhail Gorbachev [49]. Reductions in mortality were again demonstrated in 2006 when price increases led to reduced availability of alcohol [50]. Though we did not collect data in our study about the quality of alcohol consumed, cheap home-distilled spirits (“samogon”) makes up one quarter of alcohol consumed in Russia [51]. Homemade spirits are associated with increased risk to consumer health compared with manufactured spirits [52]. From 2000–2013, use of “samogon” decreased together with that of manufactured spirits, likely due to a reduction in Gross Domestic Product after 2000 and subsequent introduction of comprehensive alcohol reform policies in 2006 [52]. Rather than alcohol representing a fixed cultural practice in Russia, these findings provide optimism that policy changes at a state level may in fact reduce negative outcomes attributable to alcohol use.

In Russia there remains an emphasis on law enforcement at the expense of public health strategies for people who use substances and extreme extrajudicial policing practices such as unlawful arrest or extortion of people who inject drugs have been documented [20]. However, we observed no significant associations with environmental risk factors, including history of interactions with police and incarceration, in primary analyses. Though not statistically significant, our study observed a clinically notable association between high HIV stigma and lower mortality. This finding was unexpected and contrasts previous research indicating that stigma is a key structural barrier to care for people living with HIV in Russia [53]. Of note, in the multivariable Cox models fit in post-hoc analyses that included all the explanatory variables of interest, the conclusions were unchanged for most explanatory variables except injection drug use and selling sex, which became statistically significant. The association between injection drug use and death is an expected finding but the association between selling sex and lower risk of mortality is the opposite direction expected given that sex work is illegal in Russia and environmental risks have been documented among street-involved sex workers [54, 55]. This unexpected association may be due to confounding by covariates not included in the model. Thus further research is needed to understand the complex interplay between environmental risk factors, stigma, and other risk factors for mortality in people who use substances in Russia.

In the Cox proportional hazards model for hazardous drinking using the NIAAA definition (Table 4), and seen in all models for the explanatory variables of interest, both lower physical component score and depression were associated with mortality. Previous research has linked hazardous alcohol consumption with poorer self-reported physical health in working-aged Russian men [56]. In other settings, depression often co-occurs with alcohol use and has been shown to increase risk by way of overdose, suicide, and HIV disease progression [57, 58]. Though the effects were attenuated in the adjusted models, both low physical component score and depression remained independently associated with premature mortality. The multiple pathways that lead to premature mortality in hazardous Russian drinkers warrant further exploration.

There are several limitations to our study. First, a modest number of deaths occurred, limiting study power to detect potential associations for all factors of interest. However, the focus on short-term mortality enabled us to examine relatively proximal behaviors and conditions, which may be good opportunities for intervention. Second, the generalizability of our findings may be limited because we studied HIV-infected persons with recent hazardous alcohol use and risky sexual behaviors recruited from clinical settings in one urban area in Russia. However, it should be noted that the criteria for alcohol use and risky sex did not exclude a large proportion of screened individuals: from the 921 screened, only 110 did not meet the alcohol criteria and 134 did not meet the sex risk criteria [23]. Third, all of the explanatory variables in our study were based on self-report and may be susceptible to socially desirable reporting. However, under-reporting of risk behaviors would likely have biased results towards the null hypothesis.

## Conclusions

In summary, hazardous alcohol use represents a major modifiable risk factor for premature mortality in HIV-infected Russians who use substances [9]. Regular assessments for hazardous alcohol use and improved access to evidence-based alcohol interventions represent a critical area for expansion in the treatment of HIV-infected Russians who use substances.

## Supporting Information

S1 Appendix Survey questions in English. (PDF)

S2 Appendix Survey questions in Russian. (PDF)

S3 Appendix Minimal data set. (XLS)
