# Supplementary material for: Mortality in HIV-Infected Alcohol and Drug Users in St. Petersburg, Russia
Source: PLoS One. 2016 Nov 29;11(11):e0166539. doi: 10.1371/journal.pone.0166539 (PMC5127495; doi:10.1371/journal.pone.0166539)
Supplement: S1 Appendix — (PDF) [file pone.0166539.s001.pdf]

Healthy Relationships Intervention – Russia

BASELINE QUESTIONNAIRE

Table of Contents

Section	Instrument
A	Demographics
C	ART Medication History
D	Sex Practices
F	HIV Stigma
J	Depression (BDI II)*
L	Alcohol Use (30 day TLFB)
N	Drug Use
O	Involvement with Police
P	Overdose
Q	Suicide
T	SF12v2 Health Survey

* Sections to be self-administered

The following domains were also assessed in this study, but as no data were included in this analysis, the specific questions were not provided here.

HIV testing & HCV Diagnosis	Trauma*
Sexually Transmitted Diseases	HIV Risk*
CIDI SF: Alcohol Dependence (12 Month)	HIV Disclosure*
CIDI SF: Drug Dependence (12 Month)	Social Support
Sensation Seeking / Risk Taking	

SECTION A. DEMOGRAPHICS

"This first set of questions is related to your employment and family life."

1. What is the highest education level you have <u>completed</u>? **[SHOW CARD 1]**

   $_1$☐ < 9 grades

   $_2$☐ 11 grades

   $_3$☐ College

   $_4$☐ University

2. What has been your usual employment pattern <u>during the past 3 years</u>? **[SHOW CARD 2]**
   Note: *The answer should represent the majority of the last 3 years, not just the most recent employment status. If there are equal times for more than one category, select that which best represents the more current situation.*

   $_1$☐ Employed regularly (including official and unofficial employment)

   $_2$☐ Employed occasionally (including official and unofficial employment)

   $_3$☐ Unemployed

   $_4$☐ Student

   $_5$☐ Controlled environment

3. What is your current marital status? **[SHOW CARD 3]**

   $_1$☐ Married or living with a partner

   $_2$☐ Never married

   $_3$☐ Not currently married or not living with a partner (e.g., widowed, separated, divorced)

4. What have been your usual living arrangements in the past 3 years? **[SHOW CARD 4]**
   Note: *Choose arrangements most representative of the past 3 years. If there is an even split in time between these arrangements, choose the most recent arrangement.*

   $_1$☐ With sexual partner & children

   $_2$☐ With sexual partner alone

   $_3$☐ With children alone

   $_4$☐ With parents

   $_5$☐ With other family

   $_6$☐ With friends

   $_7$☐ Alone

   $_8$☐ Controlled environment

   $_9$☐ No stable arrangement

5. Do you live with anyone who has a current alcohol problem?

$_0$☐ No

$_1$☐ Yes

6. Do you live with anyone who uses non-prescribed drugs?
Note: *This includes illicit drugs and pharmaceutical medications that were not prescribed.*

$_0$☐ No

$_1$☐ Yes

7. Have you been in a controlled environment in the past 30 days? *(**Inpatients**: 30 days prior to this hospitalization) Select all that apply.* **[SHOW CARD 5]**

$_1$☐ No

$_1$☐ Jail

$_1$☐ Alcohol/Drug Treatment

$_1$☐ Medical Treatment

$_1$☐ Psychiatric Treatment

$_1$☐ Other: _______________ ***Note:*** *A place, theoretically, without access to drugs and alcohol.*

8. How many months were you incarcerated in your life? **[SHOW CARD 6]**

$_1$☐ Never

$_2$☐ < 1 month

$_3$☐ $\geq$ 1 month and < I year

$_4$☐ $\geq$ 1 year and <5 years

$_5$☐ $\geq$ 5 years

9*****. Have you <u>ever</u> been treated for alcohol abuse?

$_0$☐ No → *SKIP TO QUESTION 10*

$_1$☐ Yes

9a*. In the <u>past year</u> have you been treated for alcohol abuse?

$_0$☐ No

$_1$☐ Yes

10*****. Have you <u>ever</u> been treated for drug abuse?

$_0$☐ No → *SKIP TO SECTION B*

$_1$☐ Yes

10a*. In the <u>past year</u> have you been treated for drug abuse?

$_0$☐ No

1☐ Yes

*** Include detoxification, halfway houses, and in/outpatient counseling. Also include 12-step meetings (e.g., AA or NA) if the subject attended 3+ meetings within a one month period.***

SECTION C: ART MEDICATION HISTORY

"Now I'm going to ask you a few questions about antiretroviral medications."

1. Have you ever taken anti-retroviral medications for treating HIV?

(circle one)

No .... 0 → *SKIP TO SECTION D*
Yes................................ 1 → GO TO 1a

1a. If yes, when did you start? __ __/__ __ __ __ (month/year)

2. Which of the following medications have you taken for HIV?
[SHOW Chart with pill pictures and names)]

	ART Medications ever used	***Are you currently taking this drug?*** NO	YES
a.	___ ___ ___ *(enter code)*	0	1
b.	___ ___ ___ *(enter code)*	0	1
c.	___ ___ ___ *(enter code)*	0	1
d.	___ ___ ___ *(enter code)*	0	1
e.	___ ___ ___ *(enter code)*	0	1
f.	___ ___ ___ *(enter code)*	0	1
g.	___ ___ ___ *(enter code)*	0	1
h.	___ ___ ___ *(enter code)*	0	1

HIV Medication Codes

ABC = Ziagen	DRV = Prezista	TDF = Viread
ADV = Adefovir	EPZ = Epzicom	TNV = Tenofovir
APV = Agenerase	EFV = Sustiva	TPV = Aptivus
ATR = Atripla	FTC = Emtriva	TRI = Trizivir
ATV = Reyatz	IDV = Crixivan	TRU = Truvada
AZT = Retrovir	LPV = Kaletra	3TC = Epivir
COM = Combivir	NIK = Nikovir (phosphazide)	908 = Lexiva
d4T = Zerit	NFV = Viracept	OT1 = Other 1
ddC = HIVID	NVP = Viramune	OT2 = Other 2
ddI = Videx	RTV = Norvir	OT3 = Other 3
DEC = DDI EC	SQV = Invirase/Fortovase	
DLV = Rescriptor	T20 = Fuzeon	

If HIV medication code was OT1, OT2 or OT3, specify drug name:

OT1: ________________

OT2: ________________

OT3: ________________

SECTION D. SEX PRACTICES

"The next part deals with your sexual experiences and substance use. Sometimes talking about sex can be embarrassing. [Discuss confidentiality, concerns, and how the participant feels before proceeding]

The next section involves your recent sex experiences. Before we begin it is important that we agree on some words and what they mean. When I use the word "sex" I mean vaginal or anal sex.

For many of these questions, I am going to refer to things you have done or that have happened to you in the past 3-months or 90-days (for inpatients: before this hospitalization) -- that is, since (CALCULATE BACK DATE 3M). [get a calendar or date book to help the subject think back over that time]. Think back over the time since (DATE), about the places you have been, the things you have done, and the people that you have been with. Are any of the days in this period from (BACK DATE 3M) to today particularly memorable for you? Do you have any questions?

Now take a moment to think back about your sex life during the past 3 months that is, since (BACK DATE). The following questions ask about the sex you have had during that period. It is sometimes helpful to think back about dates, events, and special occasions to help you remember. Look at a calendar or date book to help you reflect back on the past 3 months. "

1. Did you have vaginal or anal sex with anyone during the past 3 months (for inpatients: before this hospitalization)?

   No ………….. 0

   Yes ………… 1

   If NO: *"So you did not have vaginal or anal sex between (BACK DATE) and today?"*

   RIGHT, I HAD NO SEX → SKIP TO SECTION E

   ACTUALLY, I DID HAVE SEX DURING THAT TIME → CONTINUE

2. How many partners have you had sex with in the past 3 months, that is, since (BACKDATE) (for inpatients, prior to this hospitalization)? __ __ __ __partners

"Now please think about the partners you have had sex with in the past 3 months, starting with the one you had sex with most recently. Please just give me their first names or initials, so that you can refer to them in the following questions. DO NOT GIVE THEIR LAST NAMES. If you can not remember their name, you can make up a name or use some other way to identify them, like assigning them a number."

How would you describe this person? **[SHOW CARD 7]**

- **MAIN or REGULAR PARTNER**: Someone who you have an ongoing relationship with and who you have sex with often- like a spouse, lover, or boyfriend or girlfriend. You can identify more than one sex partner as a main or regular partner
- **OTHER PARTNER**: Someone who you have had sex with one or more times but who is not a main or regular partner.

	FN / Initials	Select one:	
3. a. Partner 1:	__________	1☐ Main/regular	2☐ Other
b. Partner 2:	__________	1☐ Main/regular	2☐ Other
c. Partner 3:	__________	1☐ Main/regular	2☐ Other
d. Partner 4:	__________	1☐ Main/regular	2☐ Other
e. Partner 5:	__________	1☐ Main/regular	2☐ Other

<u>Partner-specific questions:</u>

Now please answer the following questions about **PARTNER 1.**

4. a. Is PARTNER 1 a man or woman?

Man …………….... 1
Woman ………….. 2

b. What is PARTNER 1's HIV status?

Negative ……….... 1
Positive ………….. 2
Don't know ….…… 3

c. In the past 3 months or since (BACK DATE), how many times did you have anal or vaginal sex with PARTNER 1? __ __ __ __

d. Of those _____ times, did you or he ever use a condom?

No ………….. 0

Yes …………. 1

e. If Yes, how many times did you or he use a condom? (note: number should be less than or equal to the total in "c" above) __ __ __ __

f. In the past 3-months (for inpatients: prior to this hospitalization), did you and Partner 1 talk about any of the following?:

1. My having HIV

No ………….. 0

Yes …………. 1

2. My partner's HIV status

No ………….. 0

Yes …………. 1

3. Using condoms

No ………….. 0

Yes …………. 1

Now, I want you to think about the past **30 days (for inpatients prior to this hospitalization)** when you had sex with PARTNER 1. Think about – where you were, what was happening, and what it was like. Ok, now please answer the following questions about that time.

g. Did you have sex with PARTNER 1 in the last 30 days (for inpatients: prior to this hospitalization)?

No ………….. 0 → SKIP TO PARTNER 2 questions

Yes …………. 1

h. Did you use alcohol or drugs before or while having sex with partner 1 at any time in the last 30 days prior to this hospitalization?

No ………….. 0 → SKIP TO PARTNER 2 questions

Yes …………. 1

i. If YES, Did you use:

(circle answer choice)	NO	YES
1. Alcohol	0	1
2. Heroin or other opioids	0	1
3. Stimulants (e.g., Jeff, ephedrine, amphetamine, methamphetamine, cocaine, crack)	0	1
4. Cannabis	0	1
5. Club drugs (e.g., ecstasy/MDMA, LSD, PCP, Special K/Ketamine)	0	1

Now please answer the following questions about **PARTNER 2.**

5. a. Is PARTNER 2 a man or woman?

Man ……………... 1
Woman ………….. 2

b. What is PARTNER 2's HIV status?

Negative ……….... 1
Positive ………….. 2
Don't know ………. 3

c. In the past 3 months or since (BACK DATE) (for inpatients: prior to this hospitalization), how many times did you have anal or vaginal sex with PARTNER 2? __ __ __ __

d. Of those _____ times, did you or he ever use a condom?

No ………….. 0
Yes ………… 1

e. If Yes, how many times did you or he use a condom? (note: number should be less than or equal to the total in "c" above) __ __ __ __

f. In the past 3-months (for inpatients: prior to this hospitalization), did you and Partner 2 talk about any of the following?:

1. My having HIV

No ………….. 0

Yes …………. 1

2. My partner’s HIV status

No ………….. 0

Yes …………. 1

3. Using condoms

No ………….. 0

Yes …………. 1

Now, I want you to think about the past **30 days (for inpatients: prior to this hospitalization)** when you had sex with PARTNER 2. Think about – where you were, what was happening, and what it was like. Ok, now please answer the following questions about that time.

g. Did you have sex with PARTNER 2 in the last 30 days (for inpatients: prior to this hospitalization)?

No ………….. 0 → SKIP TO PARTNER 3 questions

Yes …………. 1

h. Did you use alcohol or drugs before or while having sex with partner 2 at any time in the last 30 days prior to this hospitalization?

No ………….. 0 → SKIP TO PARTNER 3 questions

Yes …………. 1

i. If YES, Did you use:

**(circle answer choice)**	**NO**	**YES**
1. Alcohol	0	1
2. Heroin or other opioids	0	1
3. Stimulants (e.g., Jeff, ephedrine, amphetamine, methamphetamine, cocaine, crack)	0	1
4. Cannabis	0	1
5. Club drugs (e.g., ecstasy/MDMA, LSD, PCP, Special K/Ketamine)	0	1

Now please answer the following questions about **PARTNER 3.**

6. a. Is PARTNER 3 a man or woman?

Man …………….... 1

Woman ………….. 2

b. What is PARTNER 3's HIV status?

Negative ……….... 1

Positive ………….. 2

Don't know ….…… 3

c. In the past 3 months or since (BACK DATE) (for inpatients: prior to this hospitalization), how many times did you have anal or vaginal sex with PARTNER 3? __ __ __ __

d. Of those _____ times, did you or he ever use a condom?

No ………….. 0

Yes …………. 1

e. If Yes, how many times did you or he use a condom? (note: number should be less than or equal to the total in "c" above) __ __ __ __

f. In the past 3-months (for inpatients: prior to this hospitalization), did you and Partner 3 talk about any of the following?:

1. My having HIV

No ………….. 0

Yes …………. 1

2. My partner's HIV status

No ………….. 0

Yes …………. 1

3. Using condoms

No ………….. 0

Yes …………. 1

Now, I want you to think about the past **30 days (for inpatients: prior to this hospitalization)** when you had sex with PARTNER 3. Think about – where you were, what was happening, and what it was like. Ok, now please answer the following questions about that time.

g. Did you have sex with PARTNER 3 in the last 30 days (for inpatients: prior to this hospitalization)?

No ………….. 0 → SKIP TO PARTNER 4 questions

Yes …………. 1

h. Did you use alcohol or drugs before or while having sex at any time with partner 3 in the last 30 days prior to this hospitalization?

No ………….. 0 → SKIP TO PARTNER 4 questions

Yes …………. 1

i. If YES, Did you use:

(circle answer choice)	NO	YES
1. Alcohol	0	1
2. Heroin or other opioids	0	1
3. Stimulants (e.g., Jeff, ephedrine, amphetamine, methamphetamine, cocaine, crack)	0	1
4. Cannabis	0	1
5. Club drugs (e.g., ecstasy/MDMA, LSD, PCP, Special K/Ketamine)	0	1

Now please answer the following questions about **PARTNER 4.**

7. a. Is PARTNER 4 a man or woman?

Man ……………... 1

Woman ………….. 2

b. What is PARTNER 4's HIV status?

Negative ………... 1

Positive ………….. 2

Don't know ……… 3

c. In the past 3 months or since (BACK DATE) (for inpatients: prior to this hospitalization), how many times did you have anal or vaginal sex with PARTNER 4? __ __ __ __

d. Of those _____ times, did you or he ever use a condom?

No ………….. 0

Yes …………. 1

e. If Yes, how many times did you or he use a condom? (note: number should be less than or equal to the total in “c” above) __ __ __ __

f. In the past 3-months (for inpatients: prior to this hospitalization), did you and Partner 4 talk about any of the following?:

1. My having HIV

No ………….. 0

Yes …………. 1

2. My partner’s HIV status

No ………….. 0

Yes …………. 1

3. Using condoms

No ………….. 0

Yes …………. 1

Now, I want you to think about the past **30 days (for inpatients: prior to this hospitalization)** when you had sex with PARTNER 4. Think about – where you were, what was happening, and what it was like. Ok, now please answer the following questions about that time.

g. Did you have sex with PARTNER 4 in the last 30 days?

No ………….. 0 → SKIP TO PARTNER 5 questions

Yes …………. 1

h. Did you use alcohol or drugs before or while having sex at any time with partner 4 in the last 30 days prior to this hospitalization?

No ………….. 0 → SKIP TO PARTNER 5 questions

Yes …………. 1

i. If YES, Did you use:

(circle answer choice) NO YES

1. Alcohol	0	1
2. Heroin or other opioids	0	1
3. Stimulants (e.g., Jeff, ephedrine, amphetamine, methamphetamine, cocaine, crack)	0	1
4. Cannabis	0	1
5. Club drugs (e.g., ecstasy/MDMA, LSD, PCP, Special K/Ketamine)	0	1

Now please answer the following questions about **PARTNER 5.**

8. a. Is PARTNER 5 a man or woman?

   Man ……………..... 1

   Woman ………….. 2

   b. What is PARTNER 5's HIV status?

   Negative ……….... 1

   Positive ………….. 2

   Don't know ..……… 3

   c. In the past 3 months or since (BACK DATE) (for inpatients: prior to this hospitalization), how many times did you have anal or vaginal sex with PARTNER 5? __ __ __ __

   d. Of those _____ times, did you or he ever use a condom?

   No ………….. 0

   Yes …………. 1

   e. If Yes, how many times did you or he use a condom? (note: number should be less than or equal to the total in "c" above) __ __ __ __

   f. In the past 3-months (for inpatients: prior to this hospitalization), did you and Partner 5 talk about any of the following?:

      1. My having HIV

         No ………….. 0

         Yes …………. 1

2. My partner's HIV status

No ………….. 0

Yes …………. 1

3. Using condoms

No ………….. 0

Yes …………. 1

Now, I want you to think about the past **30 days (for inpatients: prior to this hospitalization)** when you had sex with PARTNER 5. Think about – where you were, what was happening, and what it was like. Ok, now please answer the following questions about that time.

g. Did you have sex with PARTNER 5 in the last 30 days (for inpatients: prior to this hospitalization)?

No ………….. 0 → SKIP TO CHECKPOINT below

Yes …………. 1

h. Did you use alcohol or drugs before or while having sex at any time with partner 5 in the last 30 days prior to this hospitalization?

No ………….. 0 → SKIP TO CHECKPOINT below

Yes …………. 1

i. If YES, Did you use:

**(circle answer choice)**	**NO**	**YES**
1. Alcohol	0	1
2. Heroin or other opioids	0	1
3. Stimulants (e.g., Jeff, ephedrine, amphetamine, methamphetamine, cocaine, crack)	0	1
4. Cannabis	0	1
5. Club drugs (e.g., ecstasy/MDMA, LSD, PCP, Special K/Ketamine)	0	1

CHECKPOINT: END OF PARTNER-SPECIFIC QUESTIONS.

CLARIFY WITH SUBJECT THE NUMBER OF SEX PARTNERS THEY REPORTED.

ASK THE FOLLOWING ONLY FOR THOSE PARTICIPANTS WHO REPORTED MORE THAN 5 PARTNERS; OTHERWISE SKIP TO QUESTION 13.

That completes the questions about your partners listed at the beginning. Now please answer the following questions about the other (NUMBER) you had anal or vaginal sex with in the past 3 months, that is, since (BACK DATE).

9. a. How many of these partners were women? __ __ __ __

   b. How many of these partners were men? __ __ __ __

10. In the past 3 months or since (BACK DATE), how many times did you have anal or vaginal sex with these partners? __ __ __ __Times in past 3 months

11. Of those _____ times, did you or he ever use condoms?

    No ………….. 0

    Yes ………… 1

12. If Yes, how many times did you or he use condoms? (note: number should be less than or equal to the total in "10" above) __ __ __ __

ASK EVERYONE THE FOLLOWING QUESTIONS, REGARDLESS OF HOW MANY PARTNERS THEY PREVIOUSLY REPORTED

<u>*Past 30 day questions***</u>**

Now I would like you to think about all of the sexual encounters you have had with all partners in the 30 days (for inpatients: prior to this hospitalization). **[SHOW CALENDAR TO INDICATE LAST 30 DAYS]**

13. In the 30 days (prior to this hospitalization) or since (BACK DATE), in total how many times did you have anal or vaginal sex? (note: number should be less than or equal to the combined totals they reported for all partners in past 3 months.) __ __ __

14. Of those _____ times, did you or your partner ever use a condom?

    No ………….. 0

    Yes ………… 1

15. If Yes, how many times did you or your partner use a condom? (note: number should be less than or equal to the total in "13" above) __ __ __

<u>Sex trade**</u>**

16. In the past 3 months (for inpatients: prior to this hospitalization), how often have you had sex so you could get drugs? **[SHOW CARD 8]**

    Never …………………….……….. 0

    A few times or less (1-5 times total in 3 months) ………..….. 1

    A few times each month (6-11 times total in 3 months) ………... 2

    Once or more each week (12 times total in 3 months) ………. 3

17. In the past 3 months (for inpatients: prior to this hospitalization), how often have you given drugs to someone so you could have sex with them? **[SHOW CARD 8]**

    Never …………………….……….. 0

    A few times or less (1-5 times total in 3 months) ………..….. 1

    A few times each month (6-11 times total in 3 months) ………... 2

    Once or more each week (12 times total in 3 months) ………. 3

18. In the past 3 months (for inpatients: prior to this hospitalization), how often were you **paid** money to have sex with someone? **[SHOW CARD 8]**

    Never …………………….……….. 0

    A few times or less (1-5 times total in 3 months) ………..….. 1

    A few times each month (6-11 times total in 3 months) ………... 2

    Once or more each week (12 times total in 3 months) ………. 3

19. In the past 3 months (for inpatients: prior to this hospitalization), how often did **you** give money to someone so you could have sex with them? **[SHOW CARD 8]**

    Never …………………….……….. 0

    A few times or less (1-5 times total in 3 months) ………..….. 1

    A few times each month (6-11 times total in 3 months) ………... 2

    Once or more each week (12 times total in 3 months) ………. 3

SECTION F. HIV STIGMA

Berger BE, Ferrans CE, Lashley FR. Measuring stigma in people with HIV: psychometric assessment of the HIV stigma scale. Research in Nursing and Health. 2001;24:518-529.

SECTION J. DEPRESSION

Beck AT. Beck Depression Inventory - second edition. Copyright 1996 Aaron T. Beck. Russian translation copyright 2007 Aaron T. Beck. Pearson Education, Inc.)

SECTION L. ALCOHOL USE – 30 DAY TIME-LINE FOLLOWBACK

“I’m going to ask you about your alcohol use. How many ml of each of the following alcoholic beverages (wine, beer, vodka or other spirit) did you drink on each of the following 30 days prior to your hospital admission?”

Interviewer*: use a calendar to help the subject recall drinking in the past month. For patients recruited from inpatient, record the 30 days prior to admission to the hospital.*

Beer (B)
Wine (W)
Vodka or other spirit (V)

**1**	**2**	**3**	**4**	**5**	**6**	**7**
B:	B:	B:	B:	B:	B:	B:
W:	W:	W:	W:	W:	W:	W:
V:	V:	V:	V:	V:	V:	V:
**8**	**9**	**10**	**11**	**12**	**13**	**14**
B:	B:	B:	B:	B:	B:	B:
W:	W:	W:	W:	W:	W:	W:
V:	V:	V:	V:	V:	V:	V:
**15**	**16**	**17**	**18**	**19**	**20**	**21**
B:	B:	B:	B:	B:	B:	B:
W:	W:	W:	W:	W:	W:	W:
V:	V:	V:	V:	V:	V:	V:
**22**	**23**	**24**	**25**	**26**	**27**	**28**
B:	B:	B:	B:	B:	B:	B:
W:	W:	W:	W:	W:	W:	W:
V:	V:	V:	V:	V:	V:	V:
**29**	**30**					
B:	B:					
W:	W:					
V:	V:					

SECTION N. DRUG USE

"I'm going to ask you some questions about the types of drugs you've used and how often you use them. I know that I just asked you about your prior drug use, but I'd like to quickly ask you about your use of the following drug types to help us answer a different set of questions."

A. Drugs Used

Have you ***ever*** used (injected, snorted, smoked, or pills):			If yes, have you used in the last 30 days?	
**(circle answer choice)**	**NO**	**YES**	**NO**	**YES**
1. Heroin by itself?	0	1	0	1
2. Other opioid (codeine, china white, methadone, fentanyl) by itself?	0	1	0	1
3. Jeff or ephedrine by itself?	0	1	0	1
4. Amphetamine or methamphetamine by itself?	0	1	0	1
5. Cocaine or crack by itself?	0	1	0	1
6. Heroin mixed with stimulants (Jeff, ephedrine, amphetamine, methamphetamine, cocaine or crack)?	0	1	0	1
7. Cannabis?	0	1	0	1
8. Club Drugs (ecstacy/MDMA, LSD, PCP, Special K/ Ketamine)?	0	1	0	1

B. Frequency of Use

Note: *For each substance ever used in Question 1 above, ask the following questions [i-v]. If "NO" for 1A – 1E, skip to C. Drug Injection.*

For inpatients: Begin with date 30 days prior to date of hospital admission
For outpatients: Refer to 30 days prior to today.

If none, enter "00"<br>If unsure, enter "77"<br>If refused, enter "88"	**A.**<br>Heroin or other opioids	**B.**<br>Stimulants (Jeff, ephedrine, Amphetamine, methamphetamine, cocaine, or crack)	**C.**<br>Cannabis	**D.**<br>Club drugs
**1.** How many days did you use _____ in the last 30 days?<br>(IF 00, do not ask parts **2-4**, skip to the next drug used)	___ ___	___ ___	___ ___	___ ___
**2.** How many days did you *inject* _____ in the last 30 days? (note: number should be less than or equal to the response in "1" above)	___ ___	___ ___	x	___ ___
**3.** How many *times a day* did you inject _____? (average # of injections	___ ___	___ ___	x	___ ___

/day)				
**4.** How many times a day did you use _____ *without injecting*? (daily average)	___ ___	___ ___	___ ___	___ ___

C. Drug Injection

"Sometimes people get into situations where it's difficult not to share needles. They may be in a rush to get high and starting to get sick. Or they may go across town to get drugs and not bring their tools with them, they may be shooting somewhere that isn't safe, or don't want others to see them inject."

__ __ __ 1. In the last 30 days, how many times have you injected drugs?

(if no injection use in past 30 days, skip to next section—O. Police Involvement)

__ __ __ 2. In the last 30 days, how many times (# of injections) did you inject using a needle/syringe that **you know** was sterile and new?

__ __ __ 3. In the last 30 days, how many times (# of injections) did you inject using needle/syringes that **you know** had been used by somebody else? (If zero, skip to Question 5)

Note: *Q1 should be $\geq$ Q2 + Q3. If this is not true, please clarify the responses to these questions with the subject.*

__ __ __ 4. Of the ____ times you injected after someone, how many times did you or someone else clean the needle syringes by flushing them with a liquid (e.g., bleach or water)? (note: should be less than or equal to the number reported in #3 above.)

__ __ __ 5. How many times in the last 30 days did you use a cooker/cotton/rinse water that had been used by another injector?

__ __ __ 6. How many times in the last 30 days did you fix drugs with another person, then split the drug solution (through use of the same cooker/spoon or through front or back loading)?

__ __ __ 7. How many times in the last 30 days did you let someone else use your syringe/needle after you used it?

__ __ __ 8. Of the ____ times someone used your syringe/needle after you, how many times did you or someone else flush the needle/syringe with a liquid such as bleach or water beforehand?

"Now I'm going to ask you about substances you may have used before you injected drugs in the past 30 days only." ***Note:*** *Skip drug categories if they previously said they did not use in the past 30 days. Ask everyone about alcohol use.*

	**A.**	**B.**	**C.**	**D.**	**E.**
If none, enter "00"<br>*If unsure, enter "77"*<br>*If refused, enter "88"*	Heroin or other opioids	Stimulants (Jeff or ephedrine, Amphetamine or methamphetamine, cocaine or crack)	Cannabis	Club drugs	Alcohol
9. In the last 30 days, how many times did you use ____ *before* injecting?	___ ___	___ ___	___ ___	___ ___	___ ___
10. In the last 30 days, how many times did you use ____ *before* you shared a needle/syringe (i.e., used after someone else or let someone else use your needle/syringe after you)?	___ ___	___ ___	___ ___	___ ___	___ ___

SECTION O. INVOLVEMENT WITH POLICE

1. Have you ever experienced or done the following?

		No	Yes
a.	Had syringes taken from you by the police	0	1
b.	Been arrested or detained for carrying a syringe	0	1
c.	Purchased new syringes from a pharmacy	0	1
d.	Decided not to go to the pharmacy to buy clean syringes because you were afraid of being confronted by police officers	0	1
e.	Been arrested or detained after the police “planted” syringes or drugs on you	0	1
f.	Been forced to give money to the police to keep from being arrested	0	1
g.	Been forced to have sex with a police officer	0	1

SECTION P. OVERDOSE

"The following questions ask about overdoses. We are referring to <u>*ANY overdose you may have had*</u> *including accidental and deliberate (on purpose) overdoses on illegal drugs, over the counter medications, prescription medications, or alcohol."*

1. Have you ever overdosed?

   No ………….. 0 → SKIP TO SECTION Q

   Yes ………… 1

2. How many times have you overdosed? *Enter #:* ___ ___

3. Did you ever have an overdose requiring you to go to the emergency room or requiring medical attention right away?

   No ………….. 0

   Yes ………… 1

4. Did your most recent overdose require you to go to the emergency room or require medical attention right away?

   No ………….. 0

   Yes ………… 1

5. Was your most recent overdose an attempt to kill yourself or commit suicide?

   No ………….. 0

   Yes ………… 1

6. Have you overdosed within the past 3 months?

   No ………….. 0 → SKIP TO SECTION Q

   Yes ………… 1

6a. If yes, how many times have you overdosed in the past 3 months? *Enter #:* ___ ___

SECTION Q. SUICIDE

"Now I'm going to ask some questions about emotional experiences and feelings that you may have had."

1. In your lifetime…

		Lifetime		Past 3-Months	
	**(circle answer choice)**	**NO**	**YES**	**NO**	**YES**
a.	Have you ever experienced serious thoughts of suicide?	0	1	0	1
	IF YES ASK ABOUT PAST 3 MONTHS				
b.	Have you ever attempted suicide?	0	1	0	1
	IF YES ASK ABOUT PAST 3 MONTHS				

NOTE: If no report of lifetime or recent suicide attempt , skip to Section R. If ONLY lifetime attempt, skip to Question 3. If suicide attempt in past 3 months, continue to Question 2.

2. In the past 3 months, how many times have you attempted suicide? **[SHOW CARD 11]**

Once ………………………… 1

Twice………………………… 2

Three or more times ………. 3

3. During your most recent suicide attempt, were you using…?

	**(circle answer choice)**	**NO**	**YES**
A.	Alcohol	0	1
B.	Heroin or other opioids	0	1
C.	Stimulants (e.g., Jeff, ephedrine, amphetamine, methamphetamine, cocaine, crack)	0	1
D.	Cannabis	0	1
E.	Club drugs (e.g., ecstasy/MDMA, LSD, PCP, Special K/Ketamine)	0	1

SECTION T. SF-12v2 HEALTH SURVEY

Ware JE Jr, Kosinski M, Turner-Bowker DM, Gandek B. How to score version 2 of the SF-12 health survey. Lincoln, RI: QualityMetric Incorporated, 2002.

Ware JE, Kosinski M, Turner-Bowker D, Gandek B. User's Manual for the SF-12v2™ Health Survey with a Supplement Documenting SF-12® Health Survey. Lincoln, RI: QualityMetric Incorporated, 2002.
