# Supplementary material for: Mortality in HIV-Infected Alcohol and Drug Users in St. Petersburg, Russia
Source: PLoS One. 2016 Nov 29;11(11):e0166539. doi: 10.1371/journal.pone.0166539 (PMC5127495; doi:10.1371/journal.pone.0166539)
Supplement: S2 Appendix — (PDF) [file pone.0166539.s002.pdf]

Healthy Relationships Intervention – Russia

BASELINE QUESTIONNAIRE

RUSSIAN TRANSLATION

Table of Contents

Section	Instrument
A	Demographics
C	ART Medication History
D	Sex Practices
F	HIV Stigma
J	Depression (BDI II)*
L	Alcohol Use (30 day TLFB)
N	Drug Use
O	Involvement with Police
P	Overdose
Q	Suicide
T	SF12v2 Health Survey

* Sections to be self-administered

The following domains were also assessed in this study, but as no data were included in this analysis, the specific questions were not provided here.

HIV testing & HCV Diagnosis	Trauma*
Sexually Transmitted Diseases	HIV Risk*
CIDI SF: Alcohol Dependence (12 Month)	HIV Disclosure*
CIDI SF: Drug Dependence (12 Month)	Social Support
Sensation Seeking / Risk Taking	

Раздел А. Демографические данные

1. Образование (выберите один вариант): (ПОКАЖИТЕ КАРТОЧКУ №1)
1☐ 9 классов и меньше
2☐ 11 классов
3☐ колледж/ПТУ
4☐ Университет/ВУЗ

2. Наиболее характерная занятость за последние 3 года? (ПОКАЖИТЕ КАРТОЧКУ №2)
(**Внимание**: Ответ должен отражать наиболее длительную занятость за последние 3 года, а не последний вариант. Если подходит несколько вариантов, выберите наиболее близкий к настоящему времени).
1☐ постоянная работа (включая официальную и неофициальную работу)
2☐ непостоянная работа (включая официальную и неофициальную работу)
3☐ Безработный
4☐ учащийся
5☐ В заключении, больнице, закрытой реабилитации

3. Семейное положение в настоящий момент: (ПОКАЖИТЕ КАРТОЧКУ №3)
1☐ В браке/живет с партнером
2☐ Холост, никогда не был женат/замужем
3☐ В настоящее время не состоит в браке/не живет с партнером (например, вдовец/вдова, раздельное проживание, в разводе)

4. Типичное проживание (за последние 3 года): (ПОКАЖИТЕ КАРТОЧКУ №4)
(**Внимание**: выберите варианты, наиболее отражающие типичное проживание за последние 3 года. Если было несколько одинаковых по времени вариантов, выберите наиболее близкий к настоящему времени).
1☐ С партнером и детьми
2☐ Только с партнером
3☐ Только с детьми
4☐ С родителями
5☐ С другими членами семьи
6☐ С друзьями
7☐ Один
8☐ В заключении/больнице/реабилитации
9☐ нет постоянного проживания

5. Живете ли Вы с кем-либо, имеющим алкогольные проблемы в настоящее время?
0☐ нет
1☐ да

6. Живете ли Вы с кем-либо, употребляющим наркотики или медицинские препараты, не прописанные врачом?
0☐ нет
1☐ да

7. Находились ли Вы в условиях, ограничивающих личную свободу, за последние 30 дней? (Для стационарных пациентов: за последние 30 дней до госпитализации)
Можете выбрать несколько вариантов. (ПОКАЖИТЕ КАРТОЧКУ №5)

1□ нет
2□ в тюрьме
3□ госпитализация для лечения алкоголизма или наркомании
4□ госпитализация из-за проблем с физическим здоровьем
5□ госпитализация из-за проблем с психическим здоровьем
6□ другое:______________________________ (**Внимание**: место, где теоретически невозможен доступ к алкоголю и наркотикам.)

8. Сколько месяцев Вы провели в заключении за всю жизнь? (ПОКАЖИТЕ КАРТОЧКУ №6)

1□ не был в заключении
2□ < 1месяца
3□ ≥ 1 месяца, но < I года
4□ ≥ 1 года, но < 5 лет
5□ ≥ 5 лет

9*. Лечили ли Вас **когда-нибудь** по поводу алкогольных проблем?

0□ нет →ПЕРЕЙДИТЕ К ВОПРОСУ №10
1□ да

9а*. Получали ли Вы **за последний год** лечение по поводу алкогольных проблем?

0□ нет
1□ да

10*. Лечили ли Вас **когда-нибудь** по поводу наркомании?

0□ нет →ПЕРЕЙДИТЕ К РАЗДЕЛУ Б
1□ да

10а*. Получали ли Вы **за последний год** лечение по поводу наркомании?

0□ нет
1□ да

* включая дезинтоксикацию, дома на полпути, стационарное или амбулаторное консультирование, АА и NA (если 3 и более посещения в течение 1 месяца).

Раздел В. Соблюдение приверженности к антиретровирусной терапии

1. Принимали ли Вы **когда-нибудь** антиретровирусные препараты для лечения ВИЧ?
нет.................................. 0 ПЕРЕЙДИТЕ К РАЗДЕЛУ Г
да.................................... 1

2. Какие из следующих препаратов для лечения ВИЧ Вы принимали? [ПОКАЖИТЕ КАРТУ С КАРТИНКАМИ И НАЗВАНИЯМИ]

**ABC**	**Зиаген**	**DRV**	**Презиста**	**TDF**	**Вирид**
**ADV**	**Адефовир**	**EPZ**	**Эпзиком**	**TNV**	**Тенофовир**
**APV**	**Агенераза**	**EFV**	**Сустива**	**TPV**	**Аптивус**
**ATR**	**Атрипла**	**FTC**	**Эмтрива**	**TRI**	**Тризивир**
**ATV**	**Рейац**	**IDV**	**Криксиван**	**TRU**	**Трувада**
**AZT**	**Ретровир**	**LPV**	**Калетра**	**3TC**	**Эпивир**
**COM**	**Комбивир**	**NIK**	**Никовир (фосфазид)**	**908**	**Лексива**
**d4T**	**Зерит**	**NFV**	**Вирацепт**	**OT1**	**Другой1**
**ddC**	**HIVID**	**NVP**	**Вирамун**	**OT2**	**Другой2**
**ddI**	**Видекс**	**RTV**	**Норвир**	**OT3**	**Другой3**
**DEC**	**DDI EC**	**SQV**	**Инвираза/фортоваза**		
**DLV**	**Рескриптор**	**T20**	**Фузеон**		

	Принимаете ли Вы сейчас эти препараты? **нет**	**да**
а. _____ _____ _____ *(впишите код)*	0	1
б. _____ _____ _____ *(впишите код)*	0	1
в. _____ _____ _____ *(впишите код)*	0	1
г. _____ _____ _____ *(впишите код)*	0	1
д. _____ _____ _____ *(впишите код)*	0	1
е. _____ _____ _____ *(впишите код)*	0	1
*ж.*_____ _____ _____ *(впишите код)*	0	1
з. _____ _____ _____ *(впишите код)*	0	1

Если код OT1, OT2 или OT3, укажите название препарата:
OT1: _______________
OT2: _______________
OT3: _______________

Раздел Г. Половое поведение

Вопросы последующих разделов будут касаться Вашего полового поведения и употребления наркотиков. Иногда разговор о сексуальной жизни вызывает смущение. [Обсудите конфиденциальность, беспокойство по этому поводу, что участник испытывает перед обсуждением этого раздела].
Этот раздел содержит вопросы о Вашей недавней сексуальной жизни. Перед тем как мы к нему перейдем, важно, чтобы мы договорились об употреблении одних и тех же слов и их значений. Когда я говорю о половых контактах, я имею в виду вагинальный и анальный половой контакт.
Большинство вопросов относятся к событиям прошедших трех месяцев или 90 дней для стационарных пациентов - трех месяцев или 90 дней до госпитализации), то есть начиная с (ВЫЧИСЛИТЕ ДАТУ).
Покажите участнику календарь, чтобы помочь вспомнить об этом времени.
Вспомните об этом периоде, начиная с (ДАТА), о местах, где Вы бывали, о событиях и людях, с которыми Вы встречались. Были ли за это время, начиная с (ДАТА) до сегодняшнего дня какие-то запоминающиеся события? Есть ли у Вас вопросы?
Теперь вспомните о Вашей половой жизни за прошедшие 3 месяца, начиная с (ДАТА). Эти вопросы касаются половой жизни, которую вы вели в этот период. Иногда бывает полезно вспомнить о каких-то особых событиях, датах, произошедших за это время. Посмотрите в календарь, вспомните события за прошедшие 3 месяца и скажите, пожалуйста:

1. Были ли у Вас вагинальные или анальные половые контакты за прошедшие 3 месяца (для стационарных пациентов – за прошедшие 3 месяца до госпитализации)?

Нет	0
Да	1

Если НЕТ, спросите: «Итак, у Вас не было вагинальных и анальных половых контактов с (ДАТА) до сегодняшнего дня?»
Если половых контактов не было, перейдите к следующему разделу.
Если половые контакты были, продолжайте задавать вопросы.

2. Сколько половых партнеров у Вас было за это время, с (ДАТА) до сегодняшнего дня?
_____________________________количество партнеров

Сейчас вспомните обо всех половых партнерах, с которыми у Вас были сексуальные отношения за прошедшие 3 месяца, начиная с партнера, с которым у Вас были самые недавние половые отношения. Пожалуйста, назовите мне их имена, чтобы я мог включить их в вопросы. Пожалуйста, назовите только их имена или **<u>инициалы</u>**, чтобы мы могли назвать их в последующих вопросах. НЕ НАЗЫВАЙТЕ ИХ ФАМИЛИИ. Если Вы не помните имен, можете называть их по номерам.

Как Вы можете охарактеризовать этого человека: (ПОКАЖИТЕ КАРТОЧКУ №7)
ОСНОВНОЙ ИЛИ ПОСТОЯННЫЙ ПОЛОВОЙ ПАРТНЕР: Человек, с которым у Вас имеются устойчивые отношения, с которым Вы часто вступаете в половой контакт, например, супруг(а), любовник/любовница, приятель или подруга. Вы можете считать нескольких партнеров основными или постоянными.
НЕПОСТОЯННЫЙ ПАРТНЕР: Человек, с которым у Вас были половые отношения однократно или несколько раз, но который не является постоянным половым партнером.

Инициалы	выберите один вариант	
3а. партнер 1:__________	1□ основной/постоянный	2□непостоянный
б. партнер 2:__________	1□ основной/постоянный	2□непостоянный
в. партнер 3:__________	1□ основной/постоянный	2□непостоянный
г. партнер 4:__________	1□ основной/постоянный	2□непостоянный
д. партнер 5:__________	1□ основной/постоянный	2□непостоянный

Вопросы, касающиеся каждого из партнеров:

Пожалуйста, ответьте на вопросы, касающиеся (ПАРТНЕР 1).

4. **А.** Это (ПАРТНЕР 1) мужчина или женщина?

мужчина 1
женщина 2

Б. Есть ли у (ПАРТНЕР 1) ВИЧ?

Нет 1
Да 2
Не знаю 3

В. За последние 3 месяца, начиная с (ДАТА), сколько раз у Вас были вагинальные или анальные половые контакты с (ПАРТНЕР 1)? ________

Г. Из этих _____ раз, Вы или он(а) когда-либо использовали презервативы?

Нет 0
Да 1

Д. Если да, то сколько раз Вы или он(а) использовали презервативы? ________

Е. За прошедшие 3 месяца говорили ли Вы с этим партнером о:

1. наличии у Вас ВИЧ
   Нет 0
   Да 1
2. наличии ВИЧ у Вашего партнера
   Нет 0
   Да 1
3. использовании презервативов
   Нет 0
   Да 1

Сейчас я попрошу Вас подумать о **прошедших 30 днях до госпитализации**, когда у Вас были половые контакты с (ПАРТНЕР 1). Вспомните, пожалуйста, где Вы находились, что происходило, и ответьте, пожалуйста, на вопросы, относящиеся к этому времени.

Ж. Имелись ли у Вас половые контакты с (ПАРТНЕР 1) за последние 30 дней?

Нет 0 →ПЕРЕЙДИТЕ К ВОПРОСАМ, КАСАЮЩИМСЯ ПАРТНЕРА №2
Да 1

З. Употребляли ли Вы алкоголь или наркотики перед или во время половых контактов за последние 30 дней (до госпитализации) ?

Нет 0 →ПЕРЕЙДИТЕ К ВОПРОСАМ, КАСАЮЩИМСЯ ПАРТНЕРА №2
Да 1

И. Если ДА, то какие наркотики Вы употребляли (выберите все подходящие и обведите)

		**нет**	**да**
А	алкоголь	0	1
Б	Героин и другие опиаты (кодеин, метадон, фентанил, «белый китаец»)	0	1
В	Психостимуляторы (джеф, эфедрин, амфетамин, метамфетамин, кокаин, крэк)	0	1
Г	Коноплю (гашиш, марихуану)	0	1
Д	Клубные наркотики (экстази, ЛСД, ПиСиПи, кетамин)	0	1

Сейчас я попрошу Вас ответить на вопросы, касающиеся (ПАРТНЕР 2).

5. **А.** Это (ПАРТНЕР 2) мужчина или женщина?

мужчина	1
женщина	2

Б. Есть ли у (ПАРТНЕР 2) ВИЧ?

Нет	1
Да	2
Не знаю	3

В. За последние 3 месяца, начиная с (ДАТА), сколько раз у Вас были вагинальные или анальные половые контакты с (ПАРТНЕР 2)? ________

Г. Из этих _____ раз, Вы или он(а) когда-либо использовали презервативы?

Нет	0
Да	1

Д. Если да, то сколько раз Вы или он(а) использовали презервативы? ________

Е. За прошедшие 3 месяца говорили ли Вы с этим партнером о:

1. наличии у Вас ВИЧ

Нет	0
Да	1

2. наличии ВИЧ у Вашего партнера

Нет	0
Да	1

3. использовании презервативов

Нет	0
Да	1

Сейчас я попрошу Вас подумать о **прошедших 30 днях до госпитализации**, когда у Вас были половые контакты с (ПАРТНЕР 2). Вспомните, пожалуйста, где Вы находились, что происходило, и ответьте, пожалуйста, на вопросы, относящиеся к этому времени.

Ж. Имелись ли у Вас половые контакты с (ПАРТНЕР 2) за последние 30 дней?

Нет 0 →ПЕРЕЙДИТЕ К ВОПРОСАМ, КАСАЮЩИМСЯ ПАРТНЕРА №3
Да 1

З. Употребляли ли Вы алкоголь или наркотики перед или во время половых контактов за последние 30 дней (до госпитализации) ?

Нет 0 →ПЕРЕЙДИТЕ К ВОПРОСАМ, КАСАЮЩИМСЯ ПАРТНЕРА №3
Да 1

И. Если ДА, то какие наркотики Вы употребляли (выберите все подходящие и обведите)

		**нет**	**да**
А	алкоголь	0	1
Б	Героин и другие опиаты (кодеин, метадон, фентанил, «белый китаец»)	0	1
В	Психостимуляторы (джеф, эфедрин, амфетамин, метамфетамин, кокаин, крэк)	0	1
Г	Коноплю (гашиш, марихуану)	0	1
Д	Клубные наркотики (экстази, ЛСД, ПиСиПи, кетамин)	0	1

Сейчас я попрошу Вас ответить на вопросы, касающиеся (ПАРТНЕР 3).

6. **А.** Это (ПАРТНЕР 3) мужчина или женщина?

мужчина 1
женщина 2

Б. Есть ли у (ПАРТНЕР 3) ВИЧ?

Нет 1
Да 2
Не знаю 3

В. За последние 3 месяца, начиная с (ДАТА), сколько раз у Вас были вагинальные или анальные половые контакты с (ПАРТНЕР 3)? ________

Г. Из этих _____ раз, Вы или он(а) когда-либо использовали презервативы?

Нет 0
Да 1

Д. Если да, то сколько раз Вы или он(а) использовали презервативы? ________

Е. За прошедшие 3 месяца говорили ли Вы с этим партнером о:

1. наличии у Вас ВИЧ
   Нет 0
   Да 1
2. наличии ВИЧ у Вашего партнера
   Нет 0
   Да 1
3. использовании презервативов
   Нет 0
   Да 1

Сейчас я попрошу Вас подумать о **прошедших 30 днях до госпитализации**, когда у Вас были половые контакты с (ПАРТНЕР 3). Вспомните, пожалуйста, где Вы находились, что происходило, и ответьте, пожалуйста, на вопросы, относящиеся к этому времени.

Ж. Имелись ли у Вас половые контакты с (ПАРТНЕР 3) за последние 30 дней?

Нет 0 →ПЕРЕЙДИТЕ К ВОПРОСАМ, КАСАЮЩИМСЯ ПАРТНЕРА №4
Да 1

З. Употребляли ли Вы алкоголь или наркотики перед или во время половых контактов за последние 30 дней (до госпитализации) ?

Нет 0 →ПЕРЕЙДИТЕ К ВОПРОСАМ, КАСАЮЩИМСЯ ПАРТНЕРА №4
Да 1

И. Если ДА, то какие наркотики Вы употребляли (выберите все подходящие и обведите)

		**нет**	**да**
А	алкоголь	0	1
Б	Героин и другие опиаты (кодеин, метадон, фентанил, «белый китаец»)	0	1
В	Психостимуляторы (джеф, эфедрин, амфетамин, метамфетамин, кокаин, крэк)	0	1
Г	Коноплю (гашиш, марихуану)	0	1
Д	Клубные наркотики (экстази, ЛСД, ПиСиПи, кетамин)	0	1

Сейчас я попрошу Вас ответить на вопросы, касающиеся (ПАРТНЕР 4).

7. **А.** Это (ПАРТНЕР 4) мужчина или женщина?

мужчина 1
женщина 2

Б. Есть ли у (ПАРТНЕР 4) ВИЧ?

Нет 1
Да 2
Не знаю 3

В. За последние 3 месяца, начиная с (ДАТА), сколько раз у Вас были вагинальные или анальные половые контакты с (ПАРТНЕР 4)? ________

Г. Из этих _____ раз, Вы или он(а) когда-либо использовали презервативы?

Нет 0
Да 1

Д. Если да, то сколько раз Вы или он(а) использовали презервативы? ________

Е. За прошедшие 3 месяца говорили ли Вы с этим партнером о:

1. наличии у Вас ВИЧ
   Нет 0
   Да 1
2. наличии ВИЧ у Вашего партнера
   Нет 0
   Да 1
3. использовании презервативов
   Нет 0
   Да 1

Сейчас я попрошу Вас подумать о **прошедших 30 днях до госпитализации**, когда у Вас были половые контакты с (ПАРТНЕР 4). Вспомните, пожалуйста, где Вы находились, что происходило, и ответьте, пожалуйста, на вопросы, относящиеся к этому времени.

Ж. Имелись ли у Вас половые контакты с (ПАРТНЕР 4) за последние 30 дней?

Нет 0 →ПЕРЕЙДИТЕ К ВОПРОСАМ, КАСАЮЩИМСЯ ПАРТНЕРА №5
Да 1

З. Употребляли ли Вы алкоголь или наркотики перед или во время половых контактов за последние 30 дней (до госпитализации) ?

Нет 0 →ПЕРЕЙДИТЕ К ВОПРОСАМ, КАСАЮЩИМСЯ ПАРТНЕРА №5
Да 1

И. Если ДА, то какие наркотики Вы употребляли (выберите все подходящие и обведите)

		**нет**	**да**
А	алкоголь	0	1
Б	Героин и другие опиаты (кодеин, метадон, фентанил, «белый китаец»)	0	1
В	Психостимуляторы (джеф, эфедрин, амфетамин, метамфетамин, кокаин, крэк)	0	1
Г	Коноплю (гашиш, марихуану)	0	1
Д	Клубные наркотики (экстази, ЛСД, ПиСиПи, кетамин)	0	1

Сейчас я попрошу Вас ответить на вопросы, касающиеся (ПАРТНЕР 5).

8. **А.** Это (ПАРТНЕР 5) мужчина или женщина?

мужчина 1
женщина 2

Б. Есть ли у (ПАРТНЕР 5) ВИЧ?

Нет 1
Да 2
Не знаю 3

В. За последние 3 месяца, начиная с (ДАТА), сколько раз у Вас были вагинальные или анальные половые контакты с (ПАРТНЕР 5)? ________

Г. Из этих _____ раз, Вы или он(а) когда-либо использовали презервативы?

Нет 0
Да 1

Д. Если да, то сколько раз Вы или он(а) использовали презервативы? ________

Е. За прошедшие 3 месяца говорили ли Вы с этим партнером о:

1. наличии у Вас ВИЧ
   Нет 0
   Да 1
2. наличии ВИЧ у Вашего партнера
   Нет 0
   Да 1
3. использовании презервативов
   Нет 0
   Да 1

Сейчас я попрошу Вас подумать о **прошедших 30 днях до госпитализации**, когда у Вас были половые контакты с (ПАРТНЕР 5). Вспомните, пожалуйста, где Вы находились, что происходило, и ответьте, пожалуйста, на вопросы, относящиеся к этому времени.

Ж. Имелись ли у Вас половые контакты с (ПАРТНЕР 5) за последние 30 дней?

Нет 0 →ПЕРЕЙДИТЕ К ВОПРОСАМ В КОНЦЕ РАЗДЕЛА
Да 1

З. Употребляли ли Вы алкоголь или наркотики перед или во время половых контактов за последние 30 дней (до госпитализации) ?

Нет 0 →ПЕРЕЙДИТЕ К ВОПРОСАМ В КОНЦЕ РАЗДЕЛА
Да 1

И. Если ДА, то какие наркотики Вы употребляли (выберите все подходящие и обведите)

		**нет**	**да**
А	алкоголь	0	1
Б	Героин и другие опиаты (кодеин, метадон, фентанил, «белый китаец»)	0	1
В	Психостимуляторы (джеф, эфедрин, амфетамин, метамфетамин, кокаин, крэк)	0	1
Г	Коноплю (гашиш, марихуану)	0	1
Д	Клубные наркотики (экстази, ЛСД, ПиСиПи, кетамин)	0	1

Если было 6 и более половых партнеров, заполните следующие вопросы, в противном случае перейдите к вопросам, помеченным *.**

****
ВНИМАНИЕ: ЗДЕСЬ ЗАКАНЧИВАЮТСЯ ВОПРОСЫ, КАСАЮЩИЕСЯ КАЖДОГО ИЗ ПАРТНЕРОВ. УТОЧНИТЕ У УЧАСТНИКА КОЛИЧЕСТВО ПОЛОВЫХ ПАРТНЕРОВ. СЛЕДУЮЩИЕ ВОПРОСЫ ЗАДАЮТСЯ ТОЛЬКО УЧАСТНИКАМ, ИМЕВШИМ **БОЛЕЕ** 5 ПОЛОВЫХ ПАРТНЕРОВ ЗА ПРОШЕДШИЕ 3 МЕСЯЦА. В ПРОТИВНОМ СЛУЧАЕ ПЕРЕЙДИТЕ К ВОПРОСУ №13.
****

Эти вопросы завершают перечень вопросов, относящийся к вопросам о Ваших половых партнерах. Перечисленных в начале раздела. Сейчас, пожалуйста, ответьте на вопросы, относящиеся к оставшимся (КОЛИЧЕСТВО) партнерам, с которыми Вы имели половые контакты за **последние 3 месяца**, начиная с (ДАТА).

9 а. Сколько из этих партнеров было женщин? _______
б. Сколько из этих партнеров было мужчин? _______

10. Сколько половых контактов (анальных и вагинальных) было у Вас за прошедшие 3 месяца, начиная с (ДАТА), с этими людьми? ________ количество раз за последние 3 месяца

11. Из (ЧИСЛО КОНТАКТОВ) раз, использовали ли когда-либо Вы или Ваш партнер презервативы?

Нет	0
Да	1

12. если да, то сколько раз Вы пользовались презервативами? ________

*****Вопросы, касающиеся прошедших 30 дней до госпитализации***(отвечают все пациенты)**

Сейчас я бы хотел, чтобы Вы вспомнили все половые контакты со всеми партнерами за последние 30 дней (для стационарных пациентов - до госпитализации). (ПОКАЖИТЕ КАЛЕНДАРЬ)

13. За последние 30 дней (до госпитализации), начиная с (ДАТА), сколько всего у Вас было анальных и вагинальных половых контактов**?** ________

14. Из (ЧИСЛО КОНТАКТОВ) раз, пользовались ли Вы когда-либо презервативами?

Нет	0
Да	1

15. если да, то сколько раз Вы пользовались презервативами? ________

Коммерческий секс (отвечают все пациенты)

16. Сколько раз за прошедшие 3 месяца Вы вступали в половой контакт, чтобы получить наркотики? (ПОКАЖИТЕ КАРТОЧКУ №8)

Ни разу	0
Несколько раз	1
Несколько раз в месяц	2
Один и более раз в неделю	3

17. Сколько раз за прошедшие 3 месяца Вы давали наркотики, чтобы вступить в половой контакт с этим человеком? (ПОКАЖИТЕ КАРТОЧКУ №8)

Ни разу	0
Несколько раз	1
Несколько раз в месяц	2
Один и более раз в неделю	3

18. Сколько раз за прошедшие 3 месяца **Вам** платили деньги, чтобы вступить с Вами в половой контакт? (ПОКАЖИТЕ КАРТОЧКУ №8)

Ни разу	0
Несколько раз	1
Несколько раз в месяц	2
Один и более раз в неделю	3

19. Сколько раз за прошедшие 3 месяца **Вы** платили деньги, чтобы вступить с кем-либо в половой контакт? (ПОКАЖИТЕ КАРТОЧКУ №8)

Ни разу	0
Несколько раз	1
Несколько раз в месяц	2
Один и более раз в неделю	3

Раздел E. Стигматизация ВИЧ

Berger BE, Ferrans CE, Lashley FR. Measuring stigma in people with HIV: psychometric assessment of the HIV stigma scale. Research in Nursing and Health. 2001;24:518-529.

Раздел К. Депрессия

Beck AT. Beck Depression Inventory - second edition. Copyright 1996 Aaron T. Beck. Russian translation copyright 2007 Aaron T. Beck. Pearson Education, Inc.)

Раздел М. Употребление алкоголя за последние 30 дней

Сейчас я задам вопросы об употреблении Вами алкоголя. Сколько мл каждого из перечисленных спиртных напитков (пиво (П), вино (В), крепкие напитки (К) Вы употребляли в течение каждого дня за последние 30 дней до госпитализации?

**1**	**2**	**3**	**4**	**5**	**6**	**7**
П	П	П	П	П	П	П
В	В	В	В	В	В	В
К	К	К	К	К	К	К
**8**	**9**	**10**	**11**	**12**	**13**	**14**
П	П	П	П	П	П	П
В	В	В	В	В	В	В
К	К	К	К	К	К	К
**15**	**16**	**17**	**18**	**19**	**20**	**21**
П	П	П	П	П	П	П
В	В	В	В	В	В	В
К	К	К	К	К	К	К
**22**	**23**	**24**	**25**	**26**	**27**	**28**
П	П	П	П	П	П	П
В	В	В	В	В	В	В
К	К	К	К	К	К	К
**29**	**30**	**31**				
П	П	П				
В	В	В				
К	К	К				

Раздел О. Употребление наркотиков

А. Употребление наркотиков
Сейчас я задам Вам вопросы, касающиеся употребления Вами наркотиков. Я спрошу, какие виды наркотиков Вы употребляли и как часто. Я знаю, что уже спрашивала Вас об употреблении наркотиков в прошлом, но я хотела бы еще раз быстро задать Вам вопросы об употреблении следующих видов наркотиков, Это поможет нам ответить на многие вопросы.

1. Употребляли ли Вы когда-нибудь (в виде инъекций, вдыхали, курили, глотали)

				**Если да, употребляли ли за последние 30 дней**	
		**нет**	**да**	**нет**	**да**
1	Героин (не в комбинации с другими наркотиками)	0	1	0	1
2	другие опиаты (кодеин, метадон, фентанил, «белый китаец») (не в комбинации с другими наркотиками)	0	1	0	1
3	Джеф или эфедрин (не в комбинации с другими наркотиками)	0	1	0	1
4	Амфетамины или метамфетамины (не в комбинации с другими наркотиками)	0	1	0	1
5	Кокаин или крэк (не в комбинации с другими наркотиками)	0	1	0	1
6	Комбинацию героина и стимуляторов (джеф, амфетамина, метамфетамина, эфедрина, кокаина или крэка)	0	1	0	1
7	Коноплю (гашиш, марихуану)	0	1	0	1
8	Клубные наркотики (экстази, ЛСД, ПиСиПи, кетамин)	0	1	0	1

Б. Частота употребления наркотиков

Внимание: Для каждого из наркотиков, когда-либо использовавшегося в вопросе 1, задайте следующие вопросы: (1-4). Если в вопросе 1 все ответы отрицательные, перейдите к разделу В.
Для стационарных пациентов: эти вопросы касаются периода времени за 30 дней до госпитализации.
Для амбулаторных пациентов: эти вопросы касаются последних 30 дней.

Если ответ отрицательный, впишите «00»<br>Если не уверен, впишите «77»<br>Если отказался отвечать, впишите «88»	**А. Героин и другие опиаты**	**Б. Стимуляторы (джеф, эфедрин, амфетамин, метамфетамин, кокаин, крэк)**	**В. Конопля**	**Г. Клубные наркотики**
1. Сколько дней Вы употребляли ___за последние 30 дней?<br>(если ответ отрицательный, пропустите вопросы Б-Д для данного наркотика и переходите к следующему наркотику, который употреблял пациент)	— —	— —	— —	— —
2. Сколько дней Вы употребляли ___в виде инъекций за последние 30 дней?	— —	— —	X	— —
3. Сколько раз в день Вы делали инъекции ___? (среднее число инъекций в день)	— —	— —	X	— —
4. Сколько раз в день Вы употребляли ___ не в виде инъекций? (в среднем в день)	— —	— —	— —	— —

B. Инъекционное употребление наркотиков.
Иногда люди попадают в ситуации, когда им трудно воздержаться от обмена иглами. Они могут торопиться ввести наркотик для улучшения самочувствия. Они могут приехать в другой район города за наркотиком и не иметь при себе необходимых инструментов, они могут вводить наркотик в небезопасном месте или скрываться от взглядов посторонних.

__ __ __ 1. Сколько раз за прошедшие 30 дней Вы делали инъекции наркотиков? **(если не было инъекционного употребления наркотиков за прошедшие 30 дней, перейдите к следующему разделу).**

__ __ __ 2. Из числа инъекций за прошедшие 30 дней, сколько раз Вы использовали чистые новые иглы/шприцы, которыми никто не пользовался, и **Вы об этом знали**? (укажите число инъекций)

__ __ __ 3. Из числа инъекций за прошедшие 30 дней, сколько раз Вы использовали иглы/шприцы, которыми кто-то уже пользовался, и **Вы об этом знали**? (укажите число инъекций)

Внимание: Число в вопросе №1 должно быть ≥ суммы чисел в вопросах №2 и 3. Если это не так, пожалуйста, уточните эти вопросы еще раз.

__ __ __ 4. Из числа инъекций (вопрос 3), когда Вы пользовались уже использованными иглами/шприцами, сколько раз Вы или кто-то другой промывали жидкостью (водой или отбеливателем) эти иглы/шприцы перед использованием? (укажите количество раз)

__ __ __ 5. Сколько раз за прошедшие 30 дней Вы использовали ложки/вату/воду, которые уже кто-то пользовался?

__ __ __ 6. Сколько раз за последние 30 дней Вы готовили наркотик вместе с другим человеком и делили готовый раствор, используя одни и те же ложки и шприцы для дозирования?

__ __ __ 7. Сколько раз за последние 30 дней Вы разрешали кому-либо использовать после себя шприц/иглу?

__ __ __ 8. Из числа раз, когда Вы разрешали использовать после себя иглу/шприц, сколько раз Вы промывали иглу/шприц, перед тем, как отдать их?

Сейчас я задам несколько вопросов о веществах, которые вы могли употреблять перед инъекциями наркотиков только за последние 30 дней.

Внимание: пропустите вопросы, касающиеся наркотиков, которые не употреблял участник за последние 30 дней. Обязательно задайте вопросы про употребление алкоголя.

Если ответ отрицательный, впишите «00»<br>Если не уверен, впишите «77»<br>Если отказался отвечать, впишите «88»	**А.<br>Героин и другие опиаты**	**Б.<br>Стимуляторы (джеф, эфедрин, амфетамин, метамфетамин, кокаин, крэк)**	**В.<br>Конопля**	**Г.<br>Клубные наркотики**	**Д.<br>Алкоголь**
9. Сколько раз Вы употребляли _____*перед инъекцией* наркотика за последние 30 дней?	— —	— —	— —	— —	— —
10. Сколько раз Вы употребляли _____*перед тем, как обменяться иглами/шприцами (использовали иглы/шприц после кого-то, давали другому свои иглы/шприц)* за последние 30 дней?	— —	— —	— —	— —	— —

Раздел П. Взаимодействие с правоохранительными органами

Случалось ли с Вами когда-нибудь следующее?

		**нет**	**да**
А	Милиционеры отбирали у Вас шприцы	**0**	**1**
Б	Вас арестовывали за наличие шприцев	**0**	**1**
В	Покупали новые шприцы в аптеке	**0**	**1**
Г	Решали не покупать новые шприцы в аптеке, потому что боялись, что можете быть задержаны милицией	**0**	**1**
Д	Вас арестовывали после того, как милиционеры подбрасывали Вам шприцы или наркотики	**0**	**1**
Е	Вас вынуждали давать деньги милиционерам, чтобы Вас не арестовывали	**0**	**1**
Ж	Вас принуждали вступать в половые контакты с милиционером	**0**	**1**

Раздел Р. Передозировки

Следующие вопросы касаются передозировок. Под передозировкой мы понимаем любое превышение дозы наркотика, выписанного препарата или алкоголя, включая случайное или преднамеренное.

1. Были ли у Вас **когда-либо** передозировки?
нет 0 (перейдите к следующему разделу)
да 1

2. Сколько раз у Вас были передозировки? (число раз) ___ ___

3. Приходилось ли Вам вызывать скорую помощь или другую медицинскую помощь по поводу передозировки?
нет 0
да 1

4. Приходилось ли Вам вызывать скорую помощь или другую медицинскую помощь по поводу последней передозировки?
нет 0
да 1

5. Была ли последняя передозировка попыткой суицида?
нет 0
да 1

6. Были ли у Вас передозировки за последние 3 месяца?
нет 0 (перейдите к следующему разделу)
да 1

6а. Сколько раз у Вас были передозировки за последние 3 месяца? (число раз) ___ ___

Раздел С. Суициды

Сейчас я задам Вам несколько вопросов об эмоциональных переживаниях и чувствах, которые Вы, возможно, испытывали.

1. В течение жизни…

		**В течение жизни**		**За последние 3 месяца**	
		**НЕТ**	**ДА**	**НЕТ**	**ДА**
а	Посещали ли Вас серьезные мысли о том, чтобы покончить жизнь самоубийством?	0	1	0	1
	ЕСЛИ ДА, СПРОСИТЕ, ПОЯВЛЯЛИСЬ ЛИ ЭТИ МЫСЛИ ЗА ПОСЛЕДНИЕ 3 МЕСЯЦА				
б	Предпринимали ли Вы когда-нибудь попытку самоубийства?	0	1	0	1
	ЕСЛИ ДА, СПРОСИТЕ, БЫЛИ ЛИ ПОПЫТКИ САМОУБИЙСТВА ЗА ПОСЛЕДНИЕ 3 МЕСЯЦА				

ВНИМАНИЕ: ЕСЛИ НЕ БЫЛО МЫСЛЕЙ И ПОПЫТОК В ТЕЧЕНИЕ ЖИЗНИ, ПЕРЕЙДИТЕ К СЛЕДУЮЩЕМУ РАЗДЕЛУ.
ЕСЛИ БЫЛИ ТОЛЬКО ПОПЫТКИ ИЛИ МЫСЛИ В ТЕЧЕНИЕ ЖИЗНИ, ПЕРЕЙДИТЕ К ВОПРОСУ 3.
В ОСТАЛЬНЫХ СЛУЧАЯХ ПЕРЕЙДИТЕ К ВОПРОСУ 2.

2. Сколько попыток самоубийства было за прошедшие 3 месяца? (ПОКАЖИТЕ КАРТОЧКУ № 11)

Однократно 1
Дважды 2
Три и более раз 3

3. Употребляли ли Вы во время последней попытки самоубийства…?

		**нет**	**да**
А	алкоголь	0	1
Б	героин или другие опиаты (кодеин, метадон, фентанил, «белый китаец»)	0	1
В	Стимуляторы (джеф, эфедрин, амфетамин, метамфетамин, кокаин, крэк)	0	1
Г	Коноплю (гашиш, марихуану)	0	1
Д	Клубные наркотики (экстази, ЛСД, ПиСиПи, кетамин)	0	1

Раздел Ф. SF-12v2™ Оценка здоровья

Ware JE Jr, Kosinski M, Turner-Bowker DM, Gandek B. How to score version 2 of the SF-12 health survey. Lincoln, RI: QualityMetric Incorporated, 2002.

Ware JE, Kosinski M, Turner-Bowker D, Gandek B. User's Manual for the SF-12v2™ Health Survey with a Supplement Documenting SF-12® Health Survey. Lincoln, RI: QualityMetric Incorporated, 2002.
